# Robust RFID Tag Identification

**DOI:** 10.3390/s22218406

**Published:** 2022-11-01

**Authors:** David Benedetti, Gaia Maselli

**Affiliations:** 1 Zirak, 12084 Mondovì, Italy; 2 Computer Science Department, Sapienza University of Rome, 00161 Rome, Italy

**Keywords:** RFID identification, MAC protocols, noise resiliency, sparse recovery

## Abstract

Fast and reliable identification of Radio Frequency Indentification (RFID) tags by means of anticollision (MAC) protocols has been a problem of substantial interest for more than a decade. However, improvements in identification rate have been slow, as most solutions rely on sequential approaches that try to avoid collisions, which have limited margin for performance improvement. Recently, there has been growing interest in concurrent techniques that exploit the structure of collisions to recover tag IDs. While these techniques promise substantial improvements in speed, a key question that remains unaddressed is how to deal with noise or interference that might introduce errors in the recovery process at the reader. Our goal in this paper is to consider a noisy wireless channel and add robustness to concurrent RFID identification techniques. We propose a new protocol, called CIRF (Concurrent Identification of RFids), which uses multiple antennas to add robustness to noise and leverages block sparsity-based optimization to recover EPC IDs of transmitting tags. We include fail-safe methods to handle errors that persist after the optimization stage. Extensive simulations show that CIRF achieves substantial resilience improvement in a range of very low to medium Signal-to-Noise (SNR) situations, being able to always correctly recover 99% of tags.

## 1. Introduction

RFID is considered a key technology for identification of many objects in several smart environments [1]. Fast identification of RFID tags has been a problem of substantial interest for more than a decade. The application is clear; when a user pushes a cart full of items through an RFID reader, the reader needs to quickly and accurately identify the tags, thereby precluding the need for time-consuming cashier scanning (or self-scanning) of items in the cart. There have been a large number of proposals to address the fast scanning problem; by our count, over two dozen protocols have been proposed (see [2,3,4,5,6,7,8,9,10,11,12] for a review).

While there have been many proposals, the core approach underlying these protocols is to rely on improvements to slot-based schemes. The system is centralized and tags cannot overhear each other, making options limited. Tag identification ultimately relies on a variant of ALOHA, with possible improvements in how many slots are used, inter-slot gaps, etc.

Recent research work has focused on parallel decoding techniques with the goal of improving time efficiency [13,14,15,16]. Several proposals leverage interesting developments in compressive sensing. Buzz [13] and PIP [14] are two such techniques; Buzz uses compressive sensing, while PIP uses *L*-*k* codes. While these techniques have shown the ability to greatly improve the identification rate, a major question that remains unaddressed is how to incorporate *robustness* and *reliability* into these concurrent techniques. Consider an example where a reader is attempting to identify a dozen tags that are present in front of it. The tags transmit their IDs concurrently, and the reader uses a concurrent technique (say, compressive sensing) to recover their IDs. Compressive sensing is not exact; rather, it is a multi-criterion optimization technique that attempts to identify the most likely candidate set among all possible IDs. Note that it is possible (indeed common) that the reader has no a priori knowledge of the number of tags present, or perhaps only a rough estimate of this number. In the absence of noise, this estimation is typically exact even when the number of tags is not known a priori. However, in the presence of noise this estimation process can result in false positives (i.e., identification of tags that are not present in the system) and false negatives (i.e., failure to identify tags that are present in the system). Existing approaches stop at this point, and do not tackle the problem of dealing with false positives and false negatives in noisy or high interference settings.

In this paper, we present CIRF, which adds robustness and reliability to compressive sensing-based RFID identification techniques. The CIRF protocol makes three main key contributions. First, we use multiple antennas to add robustness against noise and use a block sparsity-based approach to reduce false positives and false negatives resulting from the optimization phase. The idea is that by filtering many of the false alarms that occur in the optimization stage, there are fewer special cases to handle in the later stages, when this is more expensive. Second, we present an ID partitioning and chaining mechanism that makes the problem tractable for large network sizes. Third, we propose a mop-up protocol designed to eliminate false positives generated by the optimization phase. The protocol uses hash functions and block sparsity to achieve reliability.

Our key results are as follows:

- We show that multiple antenna-based concurrent identification results in substantial reduction of false positives and false negatives. The F-score with multi-antenna recovery is typically more than 55% higher than with single-antenna recovery.
- We demonstrate that our system can handle networks across a wide range of sizes, from small to large.
- We show that CIRF can identify tags very quickly, even in highly noisy settings; identification of twenty tags takes between 0.07 to 0.27 s, depending on the level of noise.

## 2. Existing Solutions and Their Limitations

In this section, we discuss two broad techniques that are used in RFID identification. We describe how these techniques work, as well as their pros and cons for tag identification. Specifically, we focus on two metrics: efficiency (speed) and robustness to noise. Efficiency reflects the percentage of useful time spent in identifying tags, and is often measured as the average number of slots required to identify a tag. Robustness indicates the resiliency of a protocol against noisy channels. We analyze these limits surveying the two main approaches to the tag identification problem available in the literature, namely, sequential and concurrent solutions.

### 2.1. Sequential Solutions

Tag identification has typically been addressed as an anti-collision (or arbitration) problem. There are various solutions, including ALOHA-based [3,4,5,6,7,8] and tree-based [9,10,11,12] protocols, which aim to avoid collisions in order to sequentially read tags. ALOHA protocols represent the most common approach used to coordinate access to the shared wireless channel; for example, the EPC standard is based on the ALOHA protocol [3]. Time is divided into frames of slots. Each tag randomly picks one slot, and transmits its ID when its slot arrives. If multiple tags select the same slot, they collide and have to participate in the following frame. In the case where they transmit alone, they are identified and exit protocol execution. Tree-based protocols recursively split colliding tags into two subsets until there is only one tag left in a set. Splitting happens according to an incremental binary prefix that matches tag IDs [9] or through a binary random number generator [10,11].

The main limitation of these solutions is their slowness. An optimal sequential reading process would require only one identification slot for each tag. However, the basic ALOHA protocol requires 2.7 slots on average to identify a tag, while Tree Slotted Aloha [7] decreases this factor to 2.30. Neither of the best-performing solutions [8,12] achieve this goal, as they involve collision and idle slots, which add overhead to the identification process. As an example, the BSTSA [8] protocol requires about 1.5 slots for each tag identification.

The performance of sequential protocols further degrades when considering a noisy channel, which introduces challenges to the protocol’s ability to cope with errors [17]. None of the aforementioned solutions addresses channel issues. If a tag transmission is corrupted, the reader may fail at tag identification, and the overall identification process may be delayed. The study in [17] shows that the EPC Gen2 standard anti-collision protocol (i.e., a Framed Slotted Aloha protocol) is 4.7 times slower when the SNR falls below 4 dB as compared to a noise free environment.

To deal with noisy channels, the work in [18] proposes an error resilient identification protocol which distinguishes real collisions from those caused by channel errors. The protocol follows an ALOHA scheme. When a collision is observed, the reader estimates its nature. If the collision is estimated to be real, then the reader binary splits the colliding tags. Otherwise, it asks the current tag to immediately re-transmit its ID. Although the protocol is robust against channel errors, it is inefficient, as it takes 4.3 slots per identification when the packet error rate is equal to 0.5.

### 2.2. Concurrent Solutions

A recent trend involves the use of a different approach to tag identification by exploiting collisions instead of avoiding them [19]. Early works in this direction have combined Time Division Multiple Acccess and Code Division Multiple Access approaches to simultaneously read multiple tags in a time slot [20]. Researchers have studied the use of interference cancellation in sequential solutions [21]. More recent research has focused on parallel decoding techniques, with the goal of improving time efficiency, for example, Buzz [13], PIP [14], BiGroup [15], and FlipTracer [16]. Specifically, the first two proposals address concurrent communication of tags from an algorithmic point of view, which is more related to our work, while the latter two proposals focus more on tag coding techniques, e.g., FM0 or Miller. Buzz [13] takes advantage of sparse colliding signals to optimize the identification process. The reader sends tags and estimates the number of tags, which enables tags to use a short temporary identifier (smaller than the EPC ID) to keep estimation tractable. The tags then use a random matrix with the selected temporary ID as a seed to decide which slots to transmit in. The reader observes the cumulative signals from tags, and uses a random matrix seeded by all the the possible temporary IDs to estimate the likely IDs that are present using compressive sensing. After the temporary ID of the tags have been recovered by the reader, nodes transmit their full EPC ID concurrently and the reader recovers the IDs using a belief propagation decoder. While Buzz uses sparse estimation, PIP [14] uses *L*-*k* codes for concurrent RFID identification. The reader specifies *L*, *k*, and an estimate of cardinality *m* to the tags. The tags then use these parameters to map their actual ID to a new ID of length *L* and that has *k* ones in it. Tags then transmit their *L*-*k* code concurrently, and these are decoded by the reader.

The benefit of concurrent schemes is speed. In general, techniques such asike Buzz and PIP are considerably faster, as the actual ID transfer can occur concurrently. The benefits can be significant; for example, Buzz requires roughly less than one slot per identification depending on the channel goodness, while PIP takes 0.25 slots per identification when there are 100 tags in front of the reader and assuming knowledge of all tag IDs in the system.

However, these schemes do have several drawbacks. First, Buzz relies on a bucketing mechanism to reduce the scale of compressed sensing, which is not resilient against errors. Losing a tag transmission in this phase may completely compromise its identification during compressed sensing decoding. PIP requires knowledge of the ID space, which may not be feasible in applications in which the reader is totally unaware of the tags that are present. Second, neither technique discusses how to recover from a case in which there are errors in the decoded IDs. An implicit assumption in PIP is that false positives can be discarded by simply checking the different received *L*-*k* code segments and looking for valid intersections. Similarly, Buzz does not clearly address cases in which the sparse estimation stage returns erroneous results for the recovered IDs. While dealing with errors is a standard procedure in many protocols, it is trickier in a concurrent setting, as a negative ACK is interpreted by all nodes as a failed transfer and is not directed to a specific node. Thus, reliable concurrent identification of RFIDs is a non-trivial problem.

BiGroup [15] uses the separation of collided signals in the in-phase and quadrature (IQ) domains to decode them. FlipTracer [16] aims to achieve highly reliable parallel decoding by using observed transition probabilities between signals’ combined states.

## 3. Challenges

The objective of this work is to make RFID identification through concurrent methods more robust to noise. This involves challenges at both the physical layer and at the protocol layer, as we explain below.

### 3.1. Improving Resilience to Noise

At the physical layer, the key challenge is minimizing false positives and false negatives in noisy environments. First, we consider the case of Buzz, which leverages compressive sensing for reconstruction. Compressive sensing-based reconstruction tries to optimize two objectives: to reduce reconstruction error and to increase sparsity. In noisy settings, there may be many errors in terms of both false positives and false negatives. This may occur when noise is close to the channel coefficients of certain nodes, making the nodes hard to detect, or when multiple sparse solutions appear to satisfy the reconstruction error constraints. Thus, the first challenge is improving the physical layer recovery of a candidate set of nodes that might be present in front of a reader.

### 3.2. Reliable Identification

At the next level, a key challenge in concurrent identification techniques is how the reader can provide feedback to devices in order to let them know whether their transmissions were successful, thereby enabling reliable identification of tags. This problem is easy to address in sequential systems, as the ACK is implicitly related to the immediately preceding data packet. In a concurrent system, however, a large number of nodes transmit simultaneously, and receipt of their transmissions may be unsuccessful. The challenge is for the reader to provide feedback to that subset of the nodes that are incorrectly decoded. Compounding the problem, the reader is unaware of the IDs that are present, and has no idea which it has not received successfully. A naive approach might be for the reader to echo IDs that are successfully received in a sequential manner; although the tags know which ones were not received, of course, this defeats the very purpose of concurrency, which is fast identification of tags. Thus, a key question is how to enable reliability in a concurrent setting.

### 3.3. Computational Complexity

Identification needs to occur in real time; therefore, the computational complexity needs to be sufficiently low to enable real-time identification. This is a problem specifically for concurrent approaches that use sophisticated optimization techniques for identification.

## 4. The CIRF Protocol

CIRF addresses these challenges in the following ways. To improve resilience against noise, CIRF uses multiple antennas and leverages multiple measurement vector decoding (referred to as block sparsity-based estimation) [22] to reduce false positives and false negatives. To ensure high reliability at low computational cost, CIRF partitions IDs, then uses check codes to polish (prune) the candidate ID set and hash functions to further remove false positives.

### 4.1. Basics

We consider an RFID system with multiple antennas overlapping their interrogation zones over the whole area. In our scenario, only a single antenna queries the tags, while all the antennas receive their transmissions.

When a tag transmits data, each antenna receives a signal depending on a particular amplitude and phase related to the following factors: the reader transmission power, the relative location and orientation of the tag to the reader, the material around a tag [23], and the noise level of the transmission [24]. Here, we represent the signal’s amplitude and phase as a unique channel coefficient that we assume remains constant within each round, as rounds are very short.

When multiple tags transmit, each antenna receives a complex signal that is the sum of the signals related to the set of transmitting tags. In a system with *a* antennas and *K* transmitting tags, the reader (i.e., a server) receives *a* different signals related to the same set of *K* tags [25].

### 4.2. Multiple Antennas for Resilience to Noise

The goal of the protocol is to efficiently and correctly identify variable numbers of tags in the presence of noise. Our first key contribution is the use of multiple antennas to improve resilience against noise.

We start with single antenna recovery (similar to Buzz), then describe the multiple antenna case. Single antenna recovery works as follows. Let us assume there are *K* nodes to be identified in a system of *N* nodes, where K≪N. A node with ID $n_{i}$ selects and transmits random binary numbers seeded with $n_{i}$. Although the reader does not know which IDs are present, it has a random matrix *A*, where column *i* corresponds to a node with ID *i*. Thus, *A* is an M×N random binary matrix in which each row represents a single-bit time slot and each column corresponds to the binary sequence transmitted by the tags. The elements of the column are the random bits (1 or 0) generated by a pseudorandom number generator seeded with the node ID. Thus, $A_{i,j}$ represents the *i*th bit sent by tag *j* if it is currently in the system. Transmission is simultaneous and synchronized; each node transmits the binary random sequence by sending the appropriate bit for each time slot. The goal of the recovery process is to identify which IDs are present.

This procedure can be expressed as follows:

(1) y=Ax+w

where $y=[y_{1},y_{2},\ldots ,y_{M}]$ is the vector of signals observed over *M* slots, *A* is the random matrix as described above, $x=[x_{1},x_{2},\ldots ,x_{N}]$ is the sparse vector of all the nodes that are present in the entire ID space, and $w=[w_{1},w_{2},\ldots ,w_{M}]$ is the noise vector. The *x* vector is sparse; it has only *K* non-zero entries, and the number of *K* values different from zero represents the channel coefficients of *K* transmitting nodes.

Compressive sensing provides an elegant approach to inverting Equations (1) and recovering *x* by exploiting the fact that *x* is sparse. Generally, after $O(Klog\frac{N}{K})$ measurements, *x* can be recovered successfully [26].

The caveat, however, is *w*, the noise parameter. RFID signals are weak because they are backscattered signals, and small changes in orientation or environment can impact the signal strength at the reader. In this case, sparsity-based recovery [22] provides a candidate ID set which may contain many false positives and false negatives, as no combination can perfectly recover *y* and many sparse combinations might appear as low error candidates.

The use of multiple antennas provides greater resilience against noise. In this case, the signal **Y** received by the multi-antenna reader can be represented as

(2) Y=AX+W

where **X**, **Y**, and **W** have one column for each antenna.

The key insight in recovering **X** is that it is block-sparse. In other words, the same nodes are transmitting to the different antennas, with different channel coefficients for each of the links. Thus, **X** has only a few non-zero rows in which a row element corresponds to the channel coefficients to a particular antenna.

Block sparsity can be exploited by leveraging mixed l2/l1 recovery, sometimes referred to as Multiple Measurement Vector (MMV) recovery. The key idea is to estimate the **X** matrix by formulating a convex optimization problem [22]

(3) $$\begin{array}{cccc} \; & \mathrm{minimize}\; & \; & {∥X∥}_{1,2}\\ \; & \mathrm{subject}\;\mathrm{to}\; & \; & {∥\mathrm{AX}-Y+W∥}_{F}\leq \sigma \end{array}$$

where the mixed (1,2)-norm ${∥X∥}_{1,2}$ is defined by the sum of the 2-norms of the rows of **X**, ${∥.∥}_{F}$ is the Frobenius norm, and σ is the error.

Solving the multiple antenna MMV model requires more measurements than the single antenna measurement vector case. Generally, the number of measurements required is $O(Klog\frac{N}{K}+aK)$ measurements, where *a* is the number of antennas present. Thus, the number of extra measurements required is linear in the number of nodes and antennas, which is not substantial.

### 4.3. ID Partitoning for Tractability and Hierarchical Bucketing for Scalability

A major challenge is dealing with the fact that as EPC ID is 96 bits, sparse recovery with the entire ID space would require $2^{96}$ columns in the matrix *A*, requiring substantial computational complexity.

The way CIRF makes this problem tractable is to use a mechanism of ID partitioning and chaining to aid in tractability and hierarchical bucketing, thereby achieving scalability. CIRF partitions the EPC ID into multiple sequential blocks, recovers each partition separately by applying MMV recovery, then combines the recovered partitions to obtain the full EPC ID. However, this introduces two additional challenges: (a) how to determine which partitions correspond to which node, and thereby merge them, and (b) how to eliminate false positives and negatives that result from merging the partitions.

**Figure 1:**
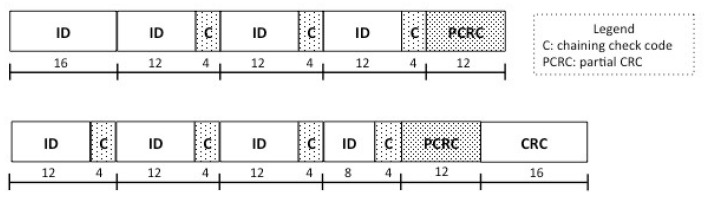
Example of EPC ID partitioning into multiple blocks and related check codes.

**Figure 2:**
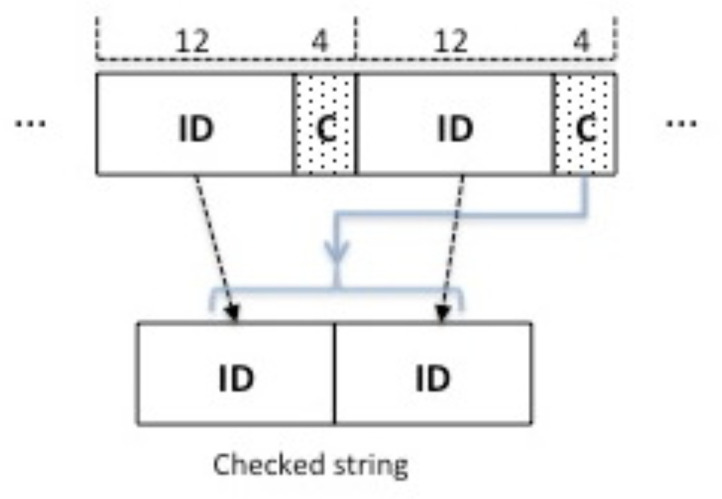
Example of using of a chaining check code.

The merging problem is addressed through check codes. The EPC ID is partitioned into a first block of *B* bits, plus multiple blocks of (*B*-*D*) bits, concatenated with *D* bits of chaining check code. Figure 1 represents an example with B=16 and D=4. Chaining check codes are related to the current and previous blocks, and allow the possible combinations of recovered tag IDs to be pruned. At each round, starting from the second one, we consider the string obtained by concatenating the previous and current ID blocks (see Figure 2). Then, we apply the $mod(2^{D})$ operation to the rest of the division between the previous block and the current one. The result is compared with the chaining code; only the blocks successfully matching the code are inserted into the candidate ID set. When the candidate set has a cardinality greater than a given threshold (e.g., 2000 candidates), we perform a Partial Cyclic Redundancy Check (PCRC) round, in which the recovered block is a Cyclic Redundancy Check (CRC) code (for example, of 12 bits), related to the ID substring recovered and merged thus far. The merging process ends with a final check performed with a global CRC code of size 16 bits, as specified in [3], which is sent in the last block and related to the entire EPC ID. This process builds EPC IDs in an incremental way; at each round *i*, the IDs in the candidate set have length roughly equal to *i* times the size of each block (*B*-*D* bits).

Note that the chaining mechanism guarantees the recovery of tags sharing a common ID substring (e.g., tags coming from the same vendor often present a common identifying prefix). If multiple tags in a round have the same ID substring, they generate the same pseudorandom sequence, and consequently are recovered as a single tag. The result is that the reader finds fewer real tags than are actually present (it may find more false positives). However, all the IDs that are found in a round are combined with all the IDs that are found in the preceding round. In this way, combinations sharing a common partition can be rebuilt. At the end of the merging process, the reader has a set of candidate IDs which includes all combinations of the recovered blocks that passed all the chaining and CRC checks.

Of course, multiple-antenna recovery requires more measurements than the single antenna case, and methods are needed to reduce the complexity of the sparse recovery system, especially when the number of tags increases. We propose a hierarchical bucketing mechanism to reduce the set of candidate node IDs, which translates to shrinking the number of columns *N* of the random matrix *A* to reduce the number of measurements *m* required to recover a reasonable set of candidate IDs, given that $m\geq Klog\left(\frac{N}{K}\right)$. Bucketing is the process of issuing a frame of slots *B* with a duration equal to the length of one bit [13]. Each slot corresponds to a bucket. Buckets are intervals of contiguous tag IDs. Each tag transmits “1” in time slot *j* if its ID falls into bucket *j*, where 1≤j≤B.

Hierarchical bucketing involves two bucketing frames. First, the ID space is divided in $B_{1}$ buckets, and each tag transmit a 1-bit in the slot corresponding to the bucket containing its ID. Then, the reader selects only the ID intervals corresponding to the buckets that received a transmission, and sends a value $B_{2}$ that the tags use together with their ID to compute a hash, the value of which is the index of the slot in which they again transmit a 1-bit. At the end of the second frame, the reader selects those IDs with hashes that lie in buckets filled with a transmission from among only those IDs considered in the previous bucket frame.

To choose $B_{1}$ and $B_{2}$, we consider a system of *K* tags and an ID space of size *S*. The expected number of columns *C* is

(4) $$C=S\cdot P_{t}^{K}\left(B_{1},B_{2}\right)=S\cdot P_{t}^{K}\left(B_{1}\right)\cdot P_{t}^{K}\left(B_{2}\right)$$

where $P_{t}^{K}\left(B\right)$ is the probability for a bucket to be filled with one or more transmissions. We exploit the fact that selections in the two frames are independent from one another.

**Figure 3:**
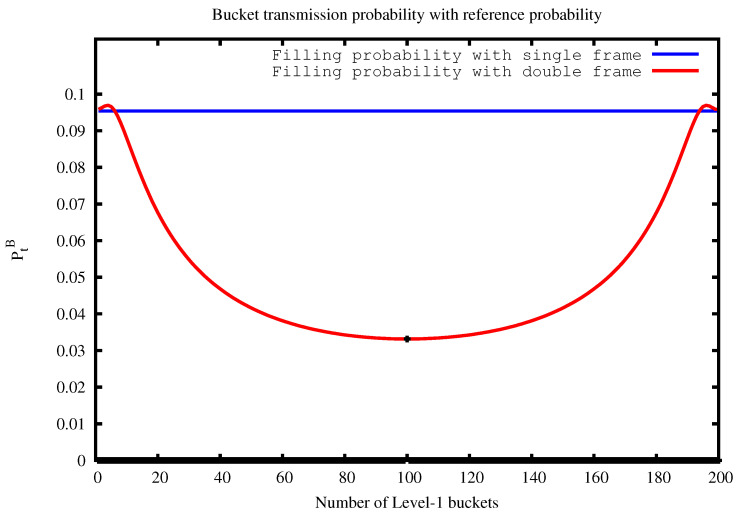
Bucket transmission probability using single and double frames.

Figure 3 shows the bucket transmission probability when K=20 and $B=B_{1}+B_{2}=200$ while varying the value of $B_{1}$. In particular, we show $P_{t}^{K}\left(B\right)$ with the blue line (i.e., the reference value) and $P_{t}^{K}\left(B_{1},B_{2}\right)$ with the red one. We can observe that the inequality $P_{t}^{K}\left(B_{1},B_{2}\right)\leq P_{t}^{K}\left(B\right)$ is strongly valid, and that this is especially true when $B_{1}\;=\;B_{2}\;=\;100$, where the red line reaches its minimum peak (the black dot in the curve). Therefore, by setting $B_{1}=B_{2}$ we obtain the minimum number of columns in the A matrix.

In addition to reducing the system complexity, we need to make the bucketing process resilient to noise. Bucketing is vulnerable to errors, as noisy channels may cause high BER, yielding two important side effects. First, because of high signal attenuation, certain 1-bit slots may appear empty from the reader side (i.e, no transmission is detected); hence, IDs that would have been selected do not appear in the random matrix, and consequently cannot be recovered. Second, because of high noise, empty bit slots may appear as non-empty from the reader side (i.e., transmission is detected), and all the IDs that lie within such slots are selected, increasing the matrix size and invalidating the main objective of bucketing. To deal with channel errors, we again exploit sparse recovery. In both bucketing phases, instead of transmitting a single 1-bit in the selected slot, tags use the generated index to seed a bit stream and send it concurrently to the reader ($A_{bucket}$ matrix). After Klog(B/K)+rK bit slots, the reader can decode the system to determine the indexes generated by the tags and the corresponding IDs. The cost of sparse recovery applied to hierarchical bucketing is provided by the number of measurements $m_{b}$ (bit slots) needed to decode the two matrixes $A_{bucket}$. As we use the same number of buckets in both frames (i.e., $B_{1}=B_{2}$), $m_{b}=2*\left(Klog\left(B_{1}/K\right)+rK\right)$.

### 4.4. Reliable Identification

The final piece of the puzzle is eliminating false positives and false negatives that persist after the above two steps. This can happen for several reasons: (a) in noisy environments, block sparsity-based recovery could miss nodes or over-generate the candidate set, and (b) while a large fraction of the errors may be eliminated by the checksums in the ID partitioning and chaining stage, errors might remain as a result of bit errors.

A naive solution to this problem would be for the reader to acknowledge every node that it has recovered successfully, allowing tags that were not present in the list to respond. However, this is highly inefficient; each such acknowledgment would need to send a checksum of the ID, which is a non-trivial number of bits compared to the original ID. Thus, such a scheme would greatly reduce the efficiency of tag identification. Ideally, we want a method that is concurrent; in other words, just as the tags can concurrently send their data to the reader, the reader should be able to concurrently send acknowledgements to all tags that have been identified thus far. The question is how to design such a concurrent acknowledgment mechanism.

While false negatives can be removed by increasing the number of measurements, false positives requires a specific mechanism. The reader mat detect tags during the optimization stage that are not actually present in the system. The key idea we use to eliminate these tags is to use hash functions and block sparsity estimation. The mechanism is based on the following assumptions:

- The tags and reader share a common hash function, which takes an EPC ID and a set of seeds as input.
- The tags and reader share a pseudo-random generator, in order to generate equal values.

At the end of the ID recovery stage, we have a set of candidates containing several FPs that must be removed. We perform a sequence of sparse recovery rounds, each having the following characteristics:

- The random matrix *A* is restricted to only the columns corresponding to the hashes of the tag IDs present in the candidate set.
- Tags use the hash function and a seed to generate the seed value used for the pseudo-random generation of the bitstream transmitted to the reader.
- The reader solves the sparse recovery system (multi-antenna) to find the real set of transmitted hashes.

After each round, the reader extracts the best ID candidates and removes from the set all the IDs that do not match any recovered hash. The process is iterated, taking a new seed in each round and producing the shrunken set as the output of the previous round. The process ends when the candidate set reaches minimum cardinality or does not change with respect to the previous round. This mechanism guarantees robustness in the removal of FPs.

## 5. Evaluation

In this section, we examine the efficiency and reliability of our CIRF identification protocol. We compare CIRF with Buzz [13], which is closest alternative to our work.

### 5.1. Implementation

Implementation of CIRF was carried out in MATLAB version R2013a. The MMV optimization stage was implemented using the SPG software package [27]. We use a ranking strategy in order to select the best candidates among the whole solution provided as outcome by the optimization software. In particular, after obtaining software output X (i.e, an estimation of tags’ attenuation factors based on different antennas), the l2-norm is computed for each row of X, then the resulting vector is listed in descending order. Finally, we consider the first K positions in this vector as our “best candidates”. To obtain an estimate of the tag cardinality *K*, the reader can use any one of the splitting processes proposed in the literature [8,13] with the cost logarithmic in K.

### 5.2. Metrics

In our performance evaluation, we focus on the F-score and efficiency metrics.

**F-score** measures the accuracy of a protocol in classifying recovered IDs, and is defined as

(5) $$\text{F-score}=\frac{2\times \mathrm{Precision}\times \mathrm{Recall}}{\mathrm{Precision}+\mathrm{Recall}}$$

where $\mathrm{Precision}=\frac{TP}{TP+FP}$, $\mathrm{Recall}=\frac{TP}{TP+FN}$, TP is the number of true positives (i.e., tags present and correctly recovered), FP is the number of false positives (i.e., tags recovered that are not present), and FN is the number of false negatives (i.e., tags that are present and not recovered).

**Efficiency** measures the time (in seconds) taken by the protocol to identify all tags, and is calculated by counting the number of single-bit slots required by the whole protocol according to the bit slot duration. When the bit rate is 40 kbps (as specified in the EPC standard [3]), a bit slot lasts 24.4 μs.

We ran Matlab simulations for K=20,50,80 tags and r=2,3,4,5 antennas. The results were obtained by averaging over 200 runs.

Our evaluation first examines the four contributions of CIRF: use of multiple antennas, partitioning over multiple rounds, hierarchical bucketing, and hashes for reliable identification. Then, we evaluate the overall protocol.

### 5.3. Evaluating Stage 1: Multiple Antenna Recovery

The first evaluation examines the costs and benefits of using multiple antennas for sparse recovery. In this evaluation, we only consider a single stage, i.e., bucketing and recovery of an ID block. As discussed in Section 4.2, the benefit of using multiple antennas is improved noise recovery, although more measurements are required to solve the block sparsity optimization.

**Figure 4:**
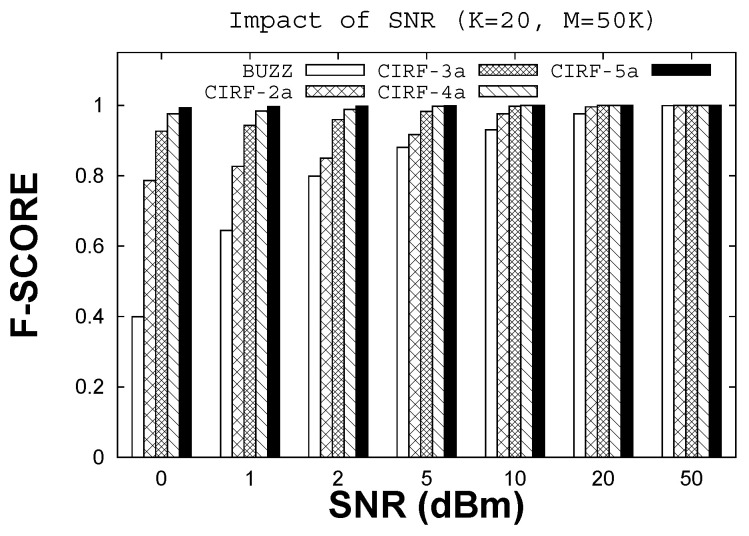
F-score with varying SNR.

To evaluate resilience to noise, we varied the SNR from 0 dBm to 50 dBm and evaluated the F-score for Buzz (single antenna) and CIRF equipped with different numbers *r* of antennas (r=2,3,4,5). We use the notation CIRF-*r*a to indicate that the CIRF protocol uses *r* antennas. The number of tags *K* is fixed at 20, and the number of measurements *M* is fixed at 1000 (50 times the number of tags), including bucketing and recovery. The results are shown in Figure 4. For high SNR, there is a small difference in F-scores for Buzz and CIRF that is not substantial; as the SNR is reduced, the difference becomes significant. At an SNR of 0dBm, the F-score for Buzz is only 0.4, whereas the F-score for CIRF with four or five antennas is more than 0.97. This is a significant difference; on average, the number of false positives with Buzz is roughly 12, whereas the number of false positives with CIRF (with five antennas is roughly 0.1, which translates into substantial savings in further rounds. The difference between Buzz and CIRF is substantial for SNRs up to about 10 dBm, after which the schemes are closer to each other. Thus, there is a significant improvement in resilience when using multiple antennas in a range of low to medium SNR situations.

**Figure 5:**
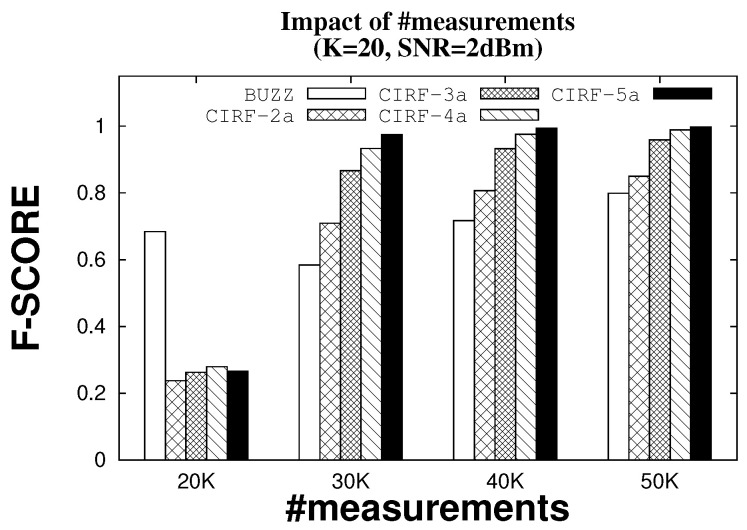
F-score with varying number of measurements (×K).

Multiple antenna recovery requires more measurements than the single antenna case; if the number of additional measurements is large, this can require quite a lot more time for identifying tags, which is undesirable. Figure 5 shows how the F-score varies as the number of measurements increases, with each plot showing the trend for a fixed number of antennas. The SNR and the number of nodes are fixed (SNR = 2 dBm, K = 20). In general, the trend showing that more antennas is better remains the case. For very small number of measurements we see a switch in ordering, and Buzz shows improvement over CIRF. This occurs only in the regime where the F-score is very low (0.67), which is not a useful operating regime. The typical operating regime would be with a number of measurements of thirty times sparsity or higher, in which case more antennas is clearly advantageous.

The above two experiments fix the number of nodes. In addition, we varied the number of nodes from 20 to 80 to determine the F-score trend while keeping the number of measurements fixed to fifty times the number of nodes and the SNR fixed to 2 dBm. The results in this case do not deviate from those seen earlier; irrespective of the number of nodes, we generally see that multiple antenna recovery is always superior to the single antenna case.

**Figure 6:**
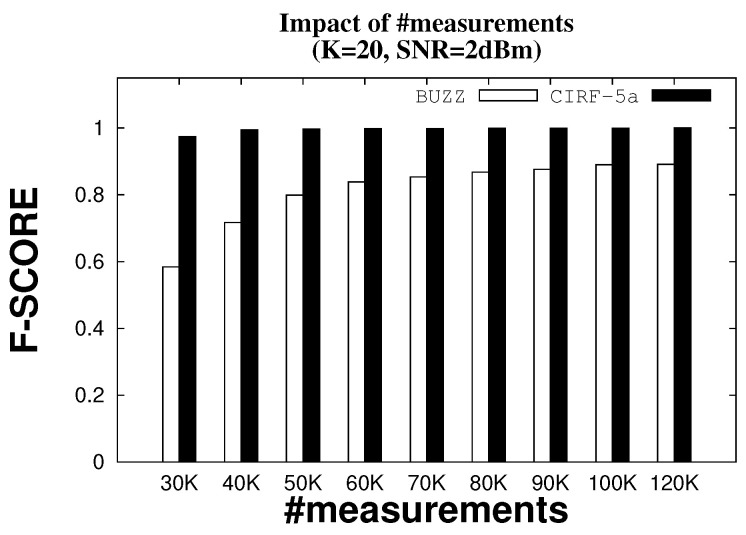
F-score with varying number of measurements.

Even with a massive increase in the number of measurements from 30 K to 120 K, Buzz fails to recover all the tag IDs. Figure 6 compares the F-scores for Buzz and CIRF while fixing the number of nodes K=20 and the SNR=2 dBm. The F-score for Buzz converges to 0.89, and never reaches full reliability (F-score = 1). However, CIRF with five antennas reaches an F-score equal to 1 at 50 K measurements.

Thus, our first set of results clearly shows the benefits of multiple antenna-based ID recovery in the presence of noise.

### 5.4. Evaluating Stage 2: ID Partitioning and Chaining

Partitioning helps to keep the computational complexity of sparse recovery in check. However, excessive partitioning reduces efficiency for several reasons: (a) more partitions means that the overall number of measurements for reconstruction increases, (b) more partitions means that the control overhead (parity bits) is larger, and (c) more partitions results in more opportunities for false positives and negatives, which in turn results in more overhead during the final recovery round.

CIRF works on blocks of 16 bits for computational tractability. Shorter blocks are not desirable, as this would require more partitions to send the entire tag ID, resulting in a higher number of false positives and false negatives in the chaining process. On the other hand, longer blocks would incur higher computational complexity. The goal is thus to maximize the number of true positives while minimizing false positives for each block of 16 bits.

Our results indicate that a good tradeoff between the number of check bits and the frequency of partial CRC is 4 bits of check code and a PCRC every 2000 candidates. Table 1 shows the simulation results of this study; we do not assume noise, as it does not have an impact on the evaluation of the mechanism. In the case with five antennas, 4 bits of check codes, and no execution of PCRC, all the *K* tags are recovered; however, a very high number of false positives (i.e., 5024) is obtained. When introducing a PCRC at every 3000 candidates, the number of false positives falls to 256. Increasing the frequency of PCRC further reduces the number of false positives; there are 27 false positives with a PCRC every 2000 candidates.

Increasing the number of bits dedicated to the chaining check code does not result in an advantage. Although it reduces the number of false positives when the PCRC is not executed (with 5 bits of check code, the number of false positives is 944), there is no performance gain when the PCRC is executed (there are 401 and 71 false positives when the PCRC is executed every 1000 and 100 candidates, respectively). Thus, 4 bits of check code and a PCRC every 2000 candidates is the best compromise in terms of bits used for ID chaining.

### 5.5. Evaluating Stage 3: Hierarchical Bucketing

The third step of the evaluation shows two significant features of CIRF bucketing, namely, noise resiliency and scalability.

**Figure 7:**
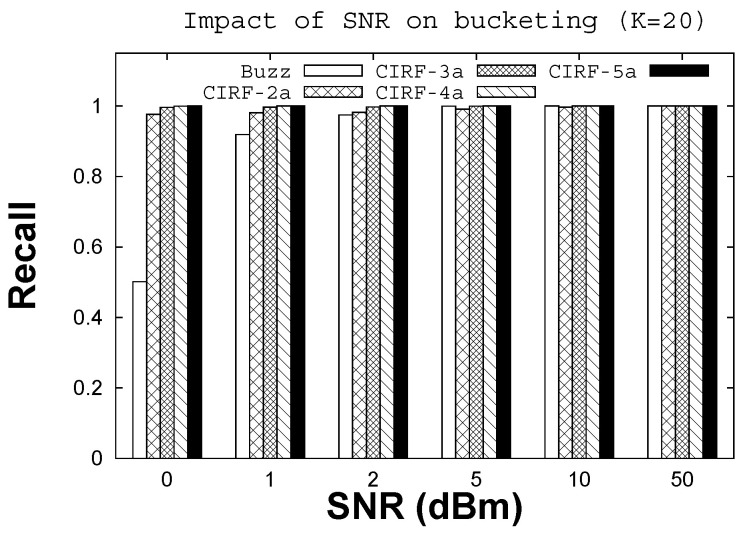
Buzz vs. CIRF bucketing.

The use of multiple antennas for sparse recovery makes bucketing resilient against noise. Figure 7 shows how recall varies when increasing the SNR. Note that recall in this case relates to buckets instead of tags; losing a bucket translates to losing one or more tags, as multiple tag IDs may fall in the same bucket. When the SNR is very low (i.e., 0 dBm), Buzz bucketing performs poorly (recall is 0.47), while CIRF bucketing shows high accuracy (recall is more than 0.99) with four or five antennas. The difference is significant, as the number of false negatives with Buzz is 10 at an SNR of 0dBm, which translates into missed tags in the identification phase (i.e., the columns corresponding to those buckets are not inserted in the A matrix). In the case of CIRF with five antennas, the number of false negatives is 0. Buzz remains inaccurate up to 2dBm, losing one bucket, while CIRF bucketing is 100% accurate. The two systems are comparable starting from the value of 5 SNR, when Buzz achieves 100% accuracy as well.

Hierarchical bucketing is highly scalable, as it keeps the number of columns in A low when the number of tags increases. Table 2 compares the number of columns selected to solve the MMV system when applying Buzz and CIRF bucketing in the absence of noise. Buzz is efficient only in the case of very small systems up to 20 tags. As the number of tags increases, the number of columns in *A* selected by Buzz grows exponentially. CIRF bucketing instead selects a candidate ID set independently of the network size, meaning that its cardinality is very close to the real number of nodes.

### 5.6. Evaluating Stage 4: Reliable Identification

**Figure 8:**
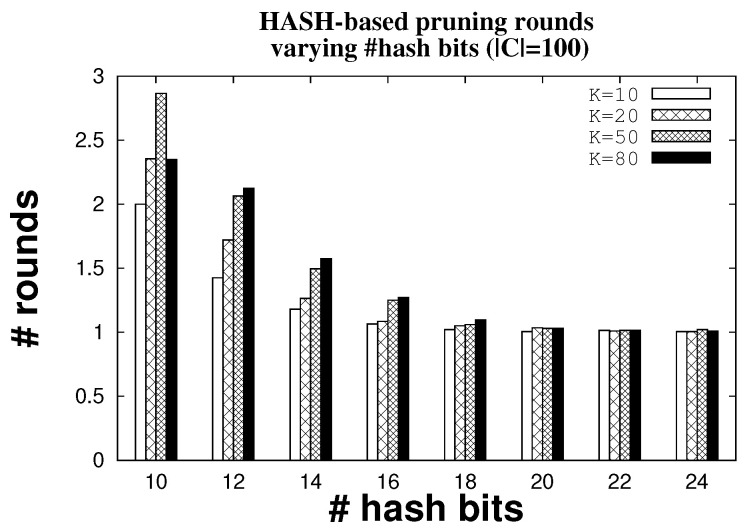
Number of rounds to remove FPs with varying hash length.

**Figure 9:**
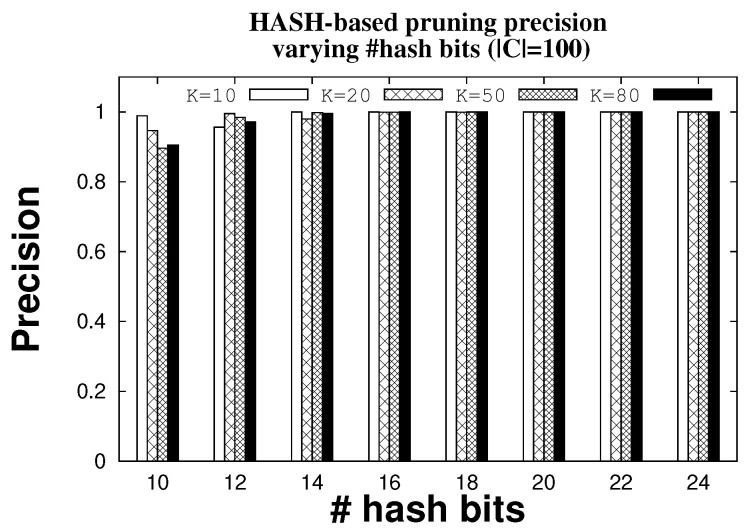
Precision of hash mechanism in removing FPs.

We use hash functions and block sparsity estimation for reliable identification. In order to better understand how many hash rounds we need to execute to mop up false positives, we plot the number (indicated by the symbol #) of rounds by varying the length of the hash functions. Figure 8 shows the trend for 10, 20, 50, and 80 tags when the candidate ID set has cardinality |C| fixed to 100 and the number of antennas is five. The mechanism is reliable. For instance, on average it takes only 1.25 rounds to remove fifty FPs when there are K = 50 tags in the system and 16 bits of hash. Increasing the length of the hash functions is one way to reduce the number of rounds; with 24 bits of hash, roughly one round is sufficient to remove all FPs. The system is precise in removing all FPs when there are at least 16 bits in the hash. Figure 9 shows that hash-based pruning is always 100% precise for hash length greater than 16 with any network size.

### 5.7. End-to-End Evaluation of CIRF

**Figure 10:**
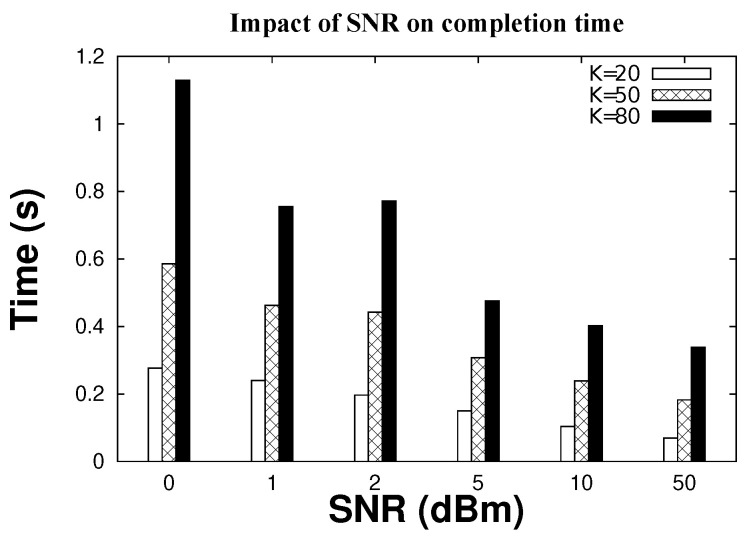
Completion time with varying SNR.

Finally, we evaluate the overall efficiency of our scheme. In terms of time, CIRF efficiency depends on the execution of multiple sparse recovery stages (ID substring recovery plus the final mop-up hash-based mechanism). Figure 10 shows the time (in seconds) for the CIRF protocol to identify all tags and remove FPs. In the case of a small network and high SNR (K = 20, SNR = 50), CIRF is able to identify all tags in less than 0.1 s. When increasing the network size to 80 tags, our system requires only 0.33 s. CIRF is efficient in the case of very high noise (SNR = 0 dBm); although it requires more measurements, the time required to recover all tag IDs only increases to 0.27 s (K = 20). Larger network sizes require more time; for example 1.13, seconds to identify 80 tags. However, this is a very short time considering the presence of very high noise (SNR = 0 dBm).

**Figure 11:**
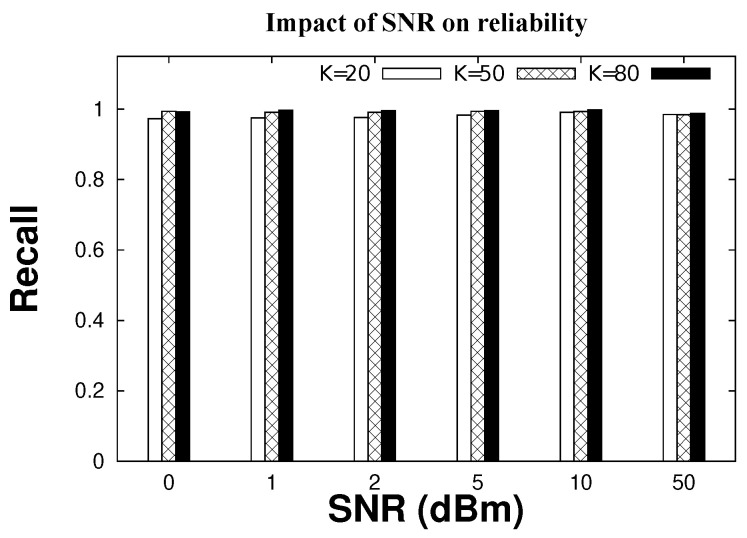
Recall for different antennas with varying SNR.

In terms of accuracy, CIRF is always able to correctly recover 99% tags when K = 50, 80; see Figure 11. This corresponds to one undetected tag every 3–4 executions, including multiple rounds for the entire ID recovery, in the case of 80 tags. The occasional failures in tag identification are due to a scenario we considered: as channel coefficients are generated randomly, it may occur that all generated coefficients are bad, leaving the reader in the worst possible condition. It is much more likely that at least one good coefficient out of five occurs in the case with five antennas. In and such case, the reader is 100% accurate.

## 6. Practical Implementation Issues

Currently, the market bases RFID tags on the EPC Gen-2 standard [3], which does not support the development of the CIRF protocol. While EPC Gen-2 is here to stay, we think it should not limit the from exploration of new designs. Indeed, CIRF allows considerably more efficient tag identification, and we think there is much to be gained from this exploration. It is possible to implement CIRF on testbed platforms, much as for Buzz. CIRF resembles Buzz in many aspects; any added complexity is mainly on the reader side. Hence, it can be implemented on the same platforms as Buzz.

Reader: It is possible to customize a USRP implementation of an EPC Gen-2 RFID reader (developed in [28]) to incorporate multiple antenna-based concurrent recovery. The traces collected by the reader can be processed on a server connected to the reader; typically, RFID systems are connected to a server that handles the data received by the reader and processes it based on the application requirements.

Tags: Moo tags [29] fit the requirements of the CIRF protocol. These are passive computational RFID tags mounting a programmable MSP430F2618 microcontroller. Moo tags have all of the components necessary for CIRF, including the ability to decode the reader’s commands, read data from memory, and transmit pseudo-random binary sequences. It is possible to implement randomization by programming different encoded traces into each Moo tag in advance. This allows the tags to transmit different data in each run without needing to be reprogrammed. At certain stages, CIRF requires the tags and the reader to share a seed. This can be communicated by the reader within the query by adding a few bits with negligible impact on communication performance; see our results on protocol latency.

Synchronization: CIRF assumes that the transmissions of tags are synchronized. Tags are naturally synchronized, as they are triggered by the reader’s signal. Tags use a digital clock to time their operations. Different clocks have different drifts, which can be corrected by having each node estimate its clock drift relative to a virtual clock maintained by the reader and compensating for the difference. As specified in [13], this can be achieved by counting the number of clock ticks between two pulses from the reader that are separated by a fixed time interval.

## 7. Conclusions

In conclusion, we present CIRF, a reliable RFID identification protocol that is: (a) fast, as a consequence of leveraging concurrency; (b) robust, by leveraging multiple antennas; and (c) reliable, by combining hash functions and block sparsity estimation. Our design has several innovative elements that have not yet been used in RFID identification, including multiple antenna-based concurrent recovery, as well as ID partitioning and chaining for computational tractability. Our results show that CIRF provides substantial robustness to noise. Furthermore, it has an advantage over other concurrent approaches such as Buzz [13] in that it leverages multiple antennas, thereby greatly reducing both false positives and false negatives. We believe that CIRF offers a path forward for practical use of compressive sensing-based techniques for concurrent RFID identification.

## Figures and Tables

**Table 1:** Number of FP for five antennas when varying the length and frequency of check codes.

Check Code Length	PCRC Frequency	FP
4	no	5024
4	3000	256
4	2000	27
5	no	944
5	2000	944
5	1000	401
5	100	71

**Table 2:** Number of columns in A.

Tags	Buzz	CIRF
10	100	101
20	397	114
50	2476	146
100	9902	193
200	39605	286
500	247522	565
1000	990077	1036

## Data Availability

Not applicable.
